# Drivers for precision livestock technology adoption: A study of factors associated with adoption of electronic identification technology by commercial sheep farmers in England and Wales

**DOI:** 10.1371/journal.pone.0190489

**Published:** 2018-01-02

**Authors:** Eliana Lima, Thomas Hopkins, Emma Gurney, Orla Shortall, Fiona Lovatt, Peers Davies, George Williamson, Jasmeet Kaler

**Affiliations:** 1 School of Veterinary Medicine and Science, University of Nottingham, Sutton Bonington Campus, Leicestershire, United Kingdom; 2 Social, Economic and Geographical Sciences, The James Hutton Institute, Craigiebuckler, Aberdeen, Scotland, United Kingdom; 3 Dunbia Ltd, Granville Industrial Estate, Dungannon, United Kingdom; University of Illinois, UNITED STATES

## Abstract

The UK is the largest lamb meat producer in Europe. However, the low profitability of sheep farming sector suggests production efficiency could be improved. Although the use of technologies such as Electronic Identification (EID) tools could allow a better use of flock resources, anecdotal evidence suggests they are not widely used. The aim of this study was to assess uptake of EID technology, and explore drivers and barriers of adoption of related tools among English and Welsh farmers. Farm beliefs and management practices associated with adoption of this technology were investigated. A total of 2000 questionnaires were sent, with a response rate of 22%. Among the respondents, 87 had adopted EID tools for recording flock information, 97 intended to adopt it in the future, and 222 neither had adopted it, neither intended to adopt it. Exploratory factor analysis (EFA) and multivariable logistic regression modelling were used to identify farmer beliefs and management practices significantly associated with adoption of EID technology. EFA identified three factors expressing farmer’s beliefs–external pressure and negative feelings, usefulness and practicality. Our results suggest farmer’s beliefs play a significant role in technology uptake. Non-adopters were more likely than adopters to believe that ‘government pressurise farmers to adopt technology’. In contrast, adopters were significantly more likely than non-adopters to see EID as practical and useful (p≤0.05). Farmers with higher information technologies literacy and intending to intensify production in the future were significantly more likely to adopt EID technology (p≤0.05). Importantly, flocks managed with EID tools had significantly lower farmer- reported flock lameness levels (p≤0.05). These findings bring insights on the dynamics of adoption of EID tools. Communicating evidence of the positive effects EID tools on flock performance and strengthening farmer’s capability in use of technology are likely to enhance the uptake of this technology in sheep farms.

## 1. Introduction

The United Kingdom (UK) is the largest lamb meat producer in Europe and the fourth largest worldwide. Despite the great size of British sheep breeding flock, sheep farming is traditionally a sector with lower profit margins than other livestock sectors such as dairy or pig farming [1–4]. Low margins coupled with heavy reliance on support payments [5] suggests there is room for increased production efficiencies in the sheep farming sector. Low record keeping traditionally seen on sheep farms is likely to be a missed opportunity on the identification of less efficiently used farm resources [5,6]. Although the use of technologies such as Electronic Identification (EID) tools simplify recording and retrieval of flock information and allow data-driven management decisions, anecdotal evidence suggest that its adoption has not been extensive, despite levy boards promotion actions in that direction. However, uptake rates have not been formally investigated in the UK.

Historically, identification of sheep in the UK was done by tattooing, piercing the ear with plastic tags or cutting notches in the external pinna. However, the introduction of an EU regulation in 2010 made Electronic Identification (EID) of all sheep mandatory, and from 2014 onwards all sheep movements had to be reported to the Animal Reporting and Movement Service (ARAMS), an animal movement database launched by the DEFRA (Department for Environment, Food & Rural Affairs). Electronic identification of individuals allows effective animal movement tracking in the event of a disease outbreak, and supports individual flock management with potential benefits with regards to labour efficiency [7]. EID identifiers (ear tags, boluses or pastern bands) contain a low radio frequency microchip with a unique identification number, which can be retrieved with an EID reader at up to 20 cm away. More advanced EID reader devices allow quick access to previous records and insertion of new data in the field. Electronic identification tag readers are an example of a “Precision Livestock Farming” (PLF) technology, which is a farm management concept developed in the mid-1980s which includes the set of tools and methods available for an efficient use of livestock resources [8–11]. EID recorded information can be used for informed decision making on several aspects of flock management, such as breeding (i.e. selecting individuals with desirable genetic traits), health (i.e. lameness, particularly with respect to culling repeatedly lame sheep), nutrition (i.e. facilitating the grouping of animals with similar body condition scores and tailoring their diet), and performance and welfare (i.e. monitoring weight gains and individual welfare outcomes) [5,12]. Despite these benefits, little is known about the use of EID technology as a management tool on sheep farms in the UK and to the authors’ knowledge there is no peer-reviewed publication on farmer’s views and opinions on this technology.

Technology acceptance and uptake is complex and influenced by a variety of factors such as socio-demographics (age, education), financial resources and farm size, with these variables having different effects on adoption. Several theories have aimed at explaining adoption of technology in the past few decades—the Theory of Reasoned Action (TRA) [13], the Technology Acceptance Model (TAM) [14], the Theory of Planned Behaviour [15–17], the Diffusion of Innovation (DOI) Theory [18], and the Technology Readiness Index [19]. These models mainly focus on technology’s ‘internal’ factors and individual perceptions related to those internal factors while ignoring any external influences (e.g. contextual, government, market). Whilst these generic models have been extensively used to explore technology adoption in sectors such as health and information systems, their usability in explaining technology adoption has not been explored widely for precision livestock farming and, specifically, investigating effect of both internal and external influences on adoption. Moreover, there are no studies on sheep farmer’s beliefs on adoption of technology in the UK.

The aims of this research were to i) explore uptake and sheep farmers beliefs about EID technology for flock management in UK, ii) explore the association between EID adoption technology and farmers beliefs and other farmer and farm characteristics, and iii) investigate the association between use of EID technology and levels of lameness on farms, as a health outcome measure.

## 2. Materials and methods

### 2.1. Study sample

A total of 2000 sheep farmers from England and Wales were sent a postal questionnaire in September 2015 enclosed with a cover letter explaining the aim of the study and data confidentiality. Commercial sheep farms supplying lamb deadweight to a major abattoir were contacted via postal mail. Farmers were invited to answer the questionnaire using the prepaid envelope enclosed with the questionnaire, and participate in a free draw with the winner receiving an iPad. To increase response rates, one reminder was sent to those farmers who had not yet answered the questionnaire.

### 2.2. Questionnaire design

The questionnaire was eight pages long and had five sections (text in S1 Questionnaire). Section 1 was designed to collect data on the farmer and the farm characteristics. It included information on years farming sheep, the farmer’s age, other enterprises on farm (i.e. beef, dairy, arable, other), self-reported information technologies (IT) knowledge, technology used at home and on farm, internet use, percentage of time spent managing sheep, number of part and full time workers on farm, and land altitude. Section 2 aimed to gather data on flock production from September 2014 to August 2015. It included questions about flock size, production information such as pregnancy scanning percentage, number of lambs sold, number of lambs retained as replacements, number of lambs retained as stores, number of ewes culled, reasons for culling sheep, and questions on whether business changes have been made in the past year and whether changes were intended over the next two years. Section 3 asked farmers to estimate flock lameness in terms of prevalence during four periods of the past year (as previous research indicated farmers can estimate prevalence levels similarly to a lameness researcher [20,21]), and frequency of use of individual treatments, including treatment with antibiotic injection, considered best practice when treating lame sheep [22,23]. Section 4 included questions on how farmers recorded information on farm, EID use and type of EID technology used by the farmer. Section 5 included 21 belief statements related to farmer’s opinions and beliefs about the use of EID for flock management. Twenty one statements were developed from Technology Acceptance Model and Technology Readiness Index constructs [24] and previous work by the researchers (Kaler and Green, 2013). Farmers were asked to answer the statements using a 5-point Likert type scale (1 = ‘disagree strongly’, 2 = ‘disagree’, 3 = ‘neither agree nor disagree’, 4 = ‘agree’ and 5 = ‘agree strongly’).

Questionnaire was pilot tested on five farmers, and improvements in the questionnaire were made accordingly before sending out to the study sample.

The study was approved by School of Veterinary Medicine and Science Ethics Committee (no: 1167 140528).

### 2.3. Data analysis

The data was analysed anonymously. The responses from the questionnaire were entered into the database software Microsoft Access and checked for errors.

Data analysis including descriptive analysis, exploratory factor analysis and multivariable logistic regression modelling was completed in Stata 14 (Statacorp, USA). Sections 1–5 were analysed descriptively using means, medians and frequencies depending on the nature of the variable. A Kruskal-Wallis test was used to investigate if there was a significant association between flock lameness levels and farmer’s use of EID technology. All usable data was used in the analysis.

#### 2.3.1. Exploratory factor analysis of farmers beliefs

An exploratory factor analysis (EFA) was performed on farmer’s belief statements. EFA is used to identify latent constructs underlying a set of related items [25]. Some checks were performed previous to the analysis. The Kaiser-Meyer-Olkin (KMO) test was done in each individual item to assess sampling adequacy (>0.5). The Bartlett test of sphericity (BS) (weighted p value x^2^ <0.05) was performed to test for the existence of relationships among variables, and the appropriateness of the correlation matrix was checked by observing a systematic covariation among the items [26]. After these checks, factor analysis followed by oblique rotation (promax) of the factors was performed to permit a degree of correlation between factors [25,26]. A scree test, based on eingenvalues of the reduced correlation matrix, was performed to aid on deciding the number of factors to be retained [25,26]. Variables with low reliability (i.e. uniqueness>0.7) and with high cross loadings were discarded [26]. The exploratory factor analysis and rotation were re-run with the selected variables, and the final solution achieved. For each set of items per factor, the Cronbach’s alpha and inter-item covariance were checked for testing for internal consistency [27,28].

#### 2.3.2. Logistic regression modelling

Two multivariable models were built to explore association between farmer beliefs (Model 1), farm/farmer characteristics (Model 2) and adoption of EID technology by farmers (outcome variable). Depending on a farmer’s reported intention to continue using or intention to adopt EID technology for farm management in the following year, they were allocated to one of the three groups. First group was composed of farmers that intended to continue using the technology (‘adopters’), a second group was composed of farmers intending to adopt it (‘intenders’) and a third group was farmers neither using nor intending to adopt it in the future (‘non-adopters’). For modelling purposes the first two groups were merged after exploring that there were no significant differences between these groups with regards to beliefs.

#### Model 1

Multivariable logistic regression was performed to model adoption/intention to adopt EID recorded information for flock management, using factors resulting from EFA as explanatory variables. For the predictor variables, each factor had scores which were computed using a non-refined method of weighted sum scores taking into consideration the strength or lack of strength of each factors' items [29].

A manual forward stepwise selection was performed [30]. P-values of ≤0.05 were retained in the model and were considered significant.

The model took the form:

Adoption/intentiontoadoptEIDrecordedinformationforflockmanagement~α+βXj+ej

Where *α* is the intercept and ~ is a logit link function, *β*Xj is series of psychosocial factors/beliefs, and ej is the residual random error that follows a binomial distribution.

#### Model 2

Multivariable logistic regression was performed to model adoption/intention to adopt EID recorded information for flock management, using farm and farmer characteristics as explanatory variables. P-values of ≤0.05 were retained in the model and were considered significant. Stepwise model building approach was used, variables with p-values ≤0.05 or considered confounders or important from previous published work were retained in the model [30]. The model took the form:

Adoption/intentiontoadoptEIDrecordedinformationforflockmanagement~α+βXj+ej

Where *α* is the intercept and ~ is a logit link function, *β*Xj is a series of explanatory variables using farm and farmer characteristics, and ej is the residual random error that follows a binomial distribution.

For Model 1 and Model 2, Pearson chi-square test was used to investigate associations between categorical variables, and non-parametric tests were used to investigate associations between continuous and categorical variables [30].

## 3. Results

A total of 439 out of 2000 questionnaires were received, generating a usable response rate of 22% (data in S1 Dataset).

### 3.1. Farmer and farm information

**Fig 1:**
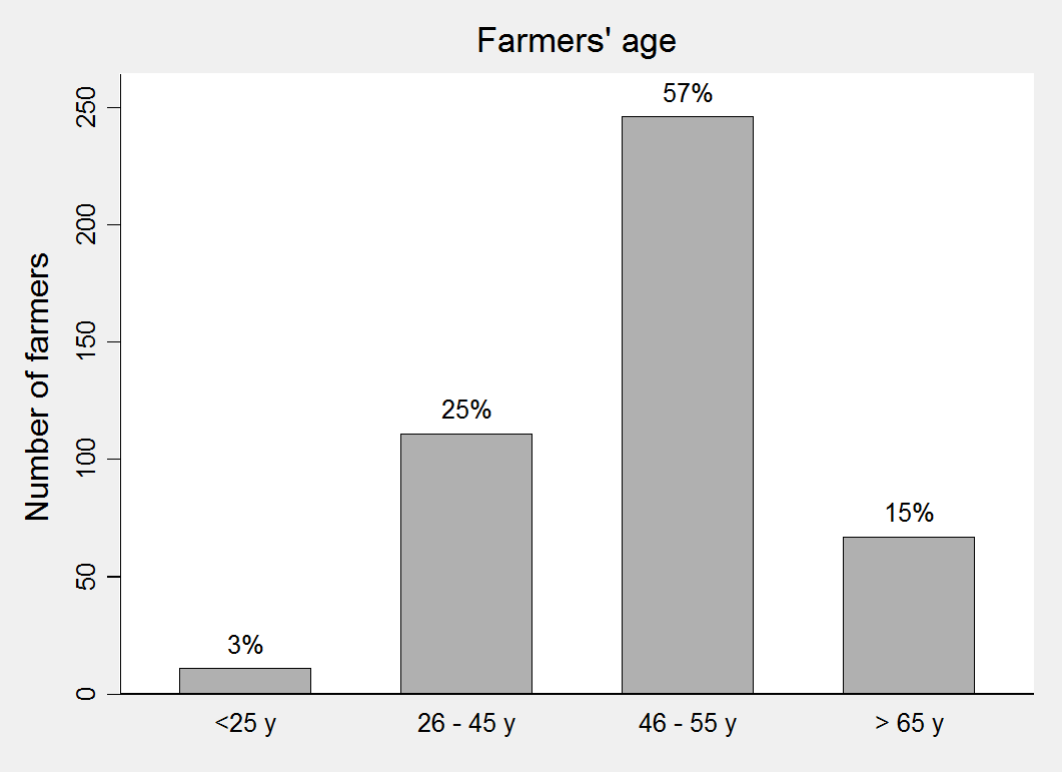
Age of farmers participating in this study (years).

**Fig 2:**
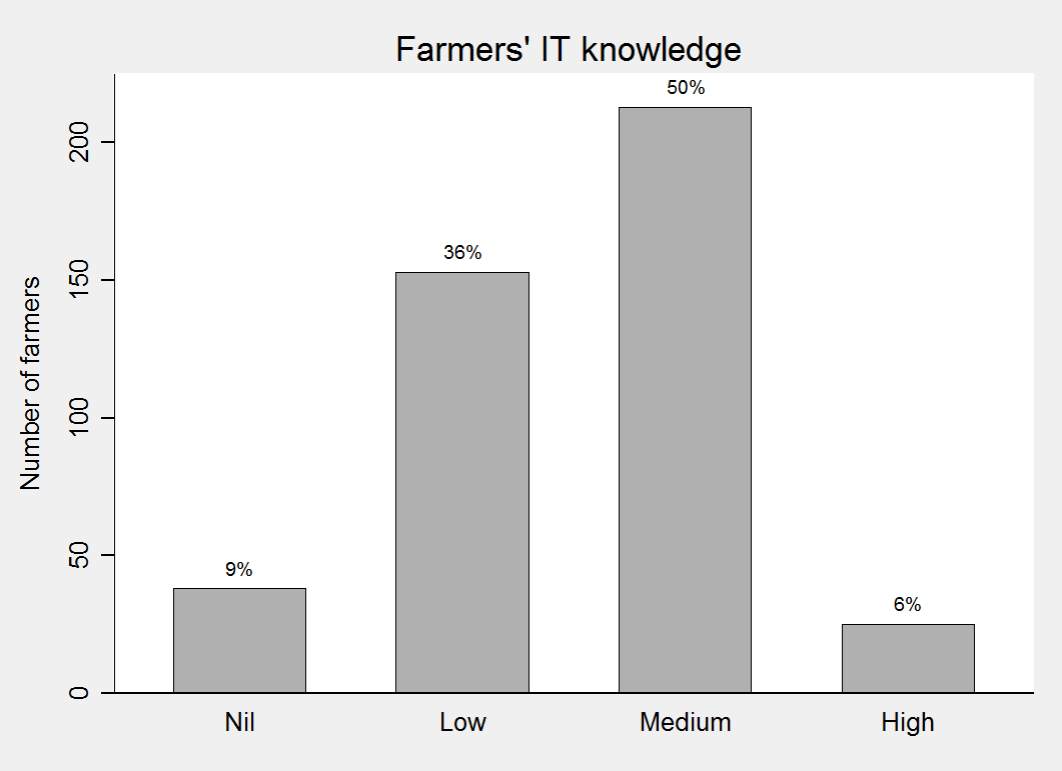
Farmers’ self-rated level of information technologies’ knowledge (nil, low, medium, or high).

The majority of farmers was between 46 and 55 years old (57%, 246/435) (Fig 1) and half of the farmers (213/429) classified their IT knowledge as “medium” (Fig 2).

**Fig 3:**
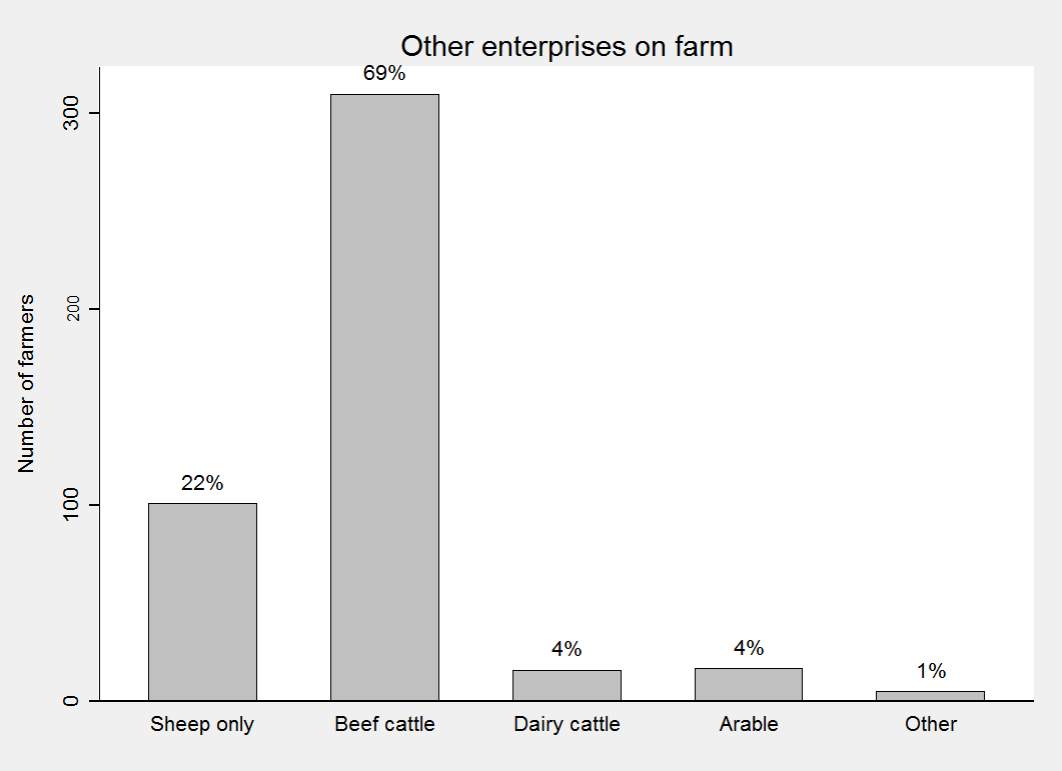
Number of farmers with other enterprises on farm (only sheep, beef cattle, dairy cattle, arable enterprise or other).

Seventy-seven per cent of farmers (327/423) used internet either for web browsing, email, or social network (twitter/ facebook), 10% of farmers reported other uses of internet, and about 13% did not use internet at all. Out of 435 farmers, approximately 46% used a smartphone (Android or iPhone) at home, but only 31% used it on farm. Forty-eight per cent (193/403) of the farms were located in the uplands, 37% in the lowlands and 15% were located in the hills. Seventy per cent (295/422) of the farms were located in Wales, while the remaining 30% were in England. Median flock size reported was 500 breeding ewes (IQR 250–850), and median scanning percentage was 160% (IQR 140–180) (363 observations). Most farmers had a beef enterprise on farm besides sheep (Fig 3).

Twenty-eight per cent (111/398) of farms hired one full time worker, and 14% and 4% of farms hired 2 and 3 full time workers respectively, during the same period. Eighty-one per cent of farmers (348/429) housed sheep at least once from September 2014 to August 2015. Median number of lambs sold was 550 (IQR 278–1000) (401 responses), median number of lambs kept for replacement was 100 (IQR 42–200) (368 responses), and median number of lambs kept as stores was 50 (IQR 0–220) (201 responses).

Regarding flock health management, ewe tooth loss was indicated by 81% (352/437) of farmers as a reason for selecting ewes for culling, followed by mastitis (70%), infertility (47%), lameness (32%), poor condition (30%) and low productivity (17%). One tenth of farmers indicated other reasons for selecting ewes for culling (i.e. prolapse, abortion (EA and Toxoplasmosis), high cull price and poor lamb prices). Twenty-six per cent (113/439) of farmers reported an intention to increase breeding flock size in the following 2 years, while 10% of farmers intended to decrease breeding flock numbers.

### 3.2. Flock lameness

Median flock lameness prevalence from September to November 2014, December 2014 to February 2015, and June to August 2015 were 5% (IQR 2–10) (368, 356 and 359 respondents respectively). From March to May 2015, median flock lameness was 5% (IQR 3–10) (361 respondents). Between September 2014 and August 2015, 15% of farmers treated a lame sheep in the same day they saw it, 31% within 3 days, 35% (153/438) within one week, 10% within 2 weeks, 2% longer than 2 weeks, and 7% never treated an individual lame sheep. Forty-six per cent (199/428) of respondents indicated they selected animals to cull based on lameness between September 2014 and August 2015.

When asked about treatment of an individual lame ewe with an antibiotic injection between September 2014 and August 2015, 42% (179/427) of farmers replied “sometimes”, 28% replied “usually”, 22% replied “always”, and 8% replied “never”.

### 3.3. Recording information on farm and use of EID technology

Seventy-three per cent (322/439) of farmers used a notebook/diary to record information on farm, 34% (148/439) of farmers used a computer, 10% (45/439) used a smartphone, 16% (70/439) used a piece of paper, and 5% (24/439) used a tablet or personal digital assistant to record flock data. Almost all (99%, 417/420) flocks used EID ear tag, with only one flock being identified with bolus, and other flock with both bolus and ear tag. Fifty-two per cent (221/423) of respondents had an EID reader on farm. Of those, handheld EID reader was the most common type, being present on 99% of farms (219/221). Four farmers had both types (static and handheld), and only two farmers had a static reader only. Forty-eight per cent (61/126) used it for managing both ewes and lambs, 40% (50/126) used it for ewes only, and 12% (15/126) used it exclusively for lamb management purposes.

A total of 87 farmers (21%) reported using EID technology for management purposes and intended to continue using the technology (‘adopters’); 97 farmers (24%) reported an intention to adopt the technology (‘intenders’) and 222 farmers (55%) reported neither using nor intending to adopt the EID technology for management purposes in future (‘non-adopters’). There was no significant difference (p>0.05) between ‘adopters’ and ‘intenders’ groups with regards to their beliefs statements, and therefore these groups were merged. Thus the resulting groups were: farmers who adopted/intended to adopt EID for flock management (n = 184), and farmers with no intention of adopting EID for flock management in the future (n = 222).

### 3.4. Farmers beliefs on data recording and results of exploratory factor analysis

The number of respondents per belief statement, and the proportion of farmers strongly agreeing, agreeing, neither agreeing or disagreeing, disagreeing and strongly disagreeing with statements on use of EID technology is presented in Table 1.

**Table 1:** Percentage of farmers strongly agreeing, agreeing, neither agreeing or disagreeing, disagreeing and strongly disagreeing with statements on use of EID technology.

Items	n	Strongly disagree	Disagree	Neither agree or disagree	Agree	Strongly agree
The ease of use of EID technology is important to my decision to use EID recording for farm management	416	4%	6%	27%	37%	26%
The time required to use EID is important to my decision to use EID recording for farm management	413	4%	6%	24%	47%	19%
The convenience of using EID is important to my decision to use EID recording for farm management	413	3%	5%	24%	45%	23%
EID assisted technology adds to the complexity of information demands placed on farmers	410	2%	9%	21%	50%	18%
There is too much pressure on farmers by the government and the market to adopt new technologies	418	2%	11%	25%	36%	26%
Current technology is not future proof, hence it is better to wait before making an investment	414	2%	17%	34%	33%	13%
Improvements in sheep health resulting from using EID are important to my decision to use EID recording for farm management	412	5%	10%	35%	36%	14%
Improvements in flock productivity resulting from using EID are important to my decision to use EID recording for farm management	411	5%	9%	33%	35%	18%
The fact EID technology should allow me to get more out of the veterinary consultation is important to my decision to use EID recording for farm management	407	5%	12%	46%	33%	4%
The fact EID technology should make it easier to receive information from the abattoir is important to my decision to use EID recording for farm management	410	2%	5%	22%	47%	24%
The fact EID technology helps with animal traceability is important to my decision to use EID recording for farm management	406	5%	8%	26%	45%	16%
The fact EID technology helps with genetic selection, genealogy and crossbreeding is important to my decision to use EID recording for farm management	407	5%	10%	41%	32%	12%
Increased technology adoption and use of precision farming is beneficial for the farming industry	413	4%	8%	25%	46%	17%
The cost of equipment is important to my decision to use EID recording for farm management.	414	3%	6%	25%	39%	27%
Adoption by other farmers is important to my decision to use EID recording for farm management.	414	11%	22%	47%	18%	2%

EFA that was run on 21 belief statements resulted in three factors. Belief statements composing each factor and correspondent loading values can be seen in Table 2. Three belief statements loaded on the first factor called here after ‘practicality’ (α = 0.921) as this included beliefs related to practical elements of technology, three statements loaded on the second factor ‘external pressure and negative feelings’ (α = 0.877) and this included combination of external pressure and negative feelings toward technology regarding feeling of added complexity or distrust, and seven statements loaded on the third factor ‘usefulness’ (α = 0.653) which included beliefs on benefits of technology.

**Table 2:** Exploratory factor analysis of 372 English and Welsh sheep farmer’s beliefs statements regarding the use of EID technology for farm management (only loadings > 0.3 are displayed).

	Exploratory Factor Analysis		
Items	Factor 1 *practicality’*	Factor 2 ‘*external pressure and negative feelings’*	Factor 3 *‘usefulness’*
The ease of use of EID technology is important to my decision to use EID recording for farm management	0.7842		
The time required to use EID is important to my decision to use EID recording for farm management	0.9376		
The convenience of using EID is important to my decision to use EID recording for farm management	0.8592		
EID assisted technology adds to the complexity of information demands placed on farmers		0.5985	
There is too much pressure on farmers by the government and the market to adopt new technologies		0.7860	
Current technology is not future proof, hence it is better to wait before making an investment		0.4986	
Improvements in sheep health resulting from using EID are important to my decision to use EID recording for farm management			0.6618
Improvements in flock productivity resulting from using EID are important to my decision to use EID recording for farm management			0.6866
The fact EID technology should allow me to get more out of the veterinary consultation is important to my decision to use EID recording for farm management			0.7625
The fact EID technology should make it easier to receive information from the abattoir is important to my decision to use EID recording for farm management			0.6938
The fact EID technology helps with animal traceability is important to my decision to use EID recording for farm management			0.6722
The fact EID technology helps with genetic selection, genealogy and crossbreeding is important to my decision to use EID recording for farm management			0.5987
Increased technology adoption and use of precision farming is beneficial for the farming industry			0.4993
Cronbach's alpha	0.921	0.877	0.653

### 3.5. Multivariable analysis of factors associated with adoption/intention to adopt EID technology for flock management

#### Model 1

All three factors (‘practicality’, ‘external pressure and negative feelings’, and ‘usefulness’) were significantly associated with adoption/intention to adopt EID technology for flock management (Table 3).

**Table 3:** Multivariable logistic regression model of psychosocial factors associated with adoption/intention to adopt EID technology for flock management (n = 350).

	O.R.	S.E.	p-value	95% C.I.
**Factor 1 –‘*practicality’***	1.18	0.09	<0.03	[1.02–1.36]
**Factor 2 – ‘*External pressure and negative feelings’***	0.73	0.06	<0.01	[0.61–0.87]
**Factor 3 *- ‘usefulness’***	1.22	0.06	<0.01	[1.10–1.35]

Logistic regression results are interpreted in terms of odds ratios (OR). The OR represents the odds that an outcome (in this case adoption of EID technology) will occur given a particular variable/factor(in this case farmer’s attitudes), compared to the odds of the outcome occurring in the absence of that variable/factor [31]. In summary, the odds ratio can be seen as a measure of effect [30]. Farmers who valued more the convenience, time and ease of use of EID technology (i.e. with higher scores on the ‘practicality’ factor) were 1.18 times (CI. 1.02–1.36) significantly more likely to adopt EID technology for management relatively to farmers with lower scores on that factor. The same effect was seen with regards to ‘usefulness’ factor, so that the more strongly farmers believed in the usefulness of the EID technology in terms of benefits related to health, productivity, veterinary consultation, abattoir feedback, traceability and breeding value, the more likely they were to adopt it (OR: 1.22 (CI 1.10–1.35)). In contrast, the more external pressure and the negative feelings (e.g. overwhelmed by complexity or scepticism in future ability of technology) farmers felt towards the technology the less likely they were to adopt EID technology (OR: 0.73, CI: 0.61–0.87) (Table 3). All three factors were significantly correlated with each other with factor 1 and 3 positively associated and both negatively associated with factor 2.

#### Model 2

Farm or farmer characteristics significantly associated with adoption or intention to adopt EID recorded information for flock management were: IT knowledge, use of smartphone to record information on farm, intention to intensify production in the next two years, time spent with the flock from September 2014 to August 2015, and always using an antibiotic injection to treat lame ewes from September 2014 to August 2015 (Table 4).

**Table 4:** Multivariable logistic regression model of farmer or farm factors associated with adoption/intention to adopt EID technology for flock management (n = 351).

	n	O.R.	S.E.	p-value	95% CI
IT knowledge–nil	35				
IT knowledge—low	145	3.88	2.25	0.02	[1.24–12.10]
IT knowledge—medium	196	5.24	3.04	0.01	[1.69–16.32]
IT knowledge—high	23	13.43	11.46	0.01	[2.52–71.55]
Do not use a smartphone to record information on farm	362				
Use a smartphone to record information on farm	44	3.69	1.90	0.01	[1.36–10.13]
Proportion of work time spent managing sheep	394	1.01	0.005	0.04	[1.00–1.02]
Do not intend to intensify production in the next two years	221				
Intend to intensify production in the next two years	183	5.10	2.51	0.01	[1.94–13.83]
Never use best practice to treat lame sheep	31				
Sometimes use best practice to treat lame sheep	165	2.22	1.16	0.12	[0.80–6.16]
Usually use best practice to treat lame sheep	114	1.54	0.82	0.42	[0.54–4.37]
Always use best practice to treat lame sheep	90	2.97	1.60	0.04	[1.034–8.55]
Flock size	400	1.00	0.20	0.84	[0.99–1.00]
Age category– 25 or less years old	10				
Age category–from 26 to 35 years old	41	0.37	0.35	0.23	[0.06–2.40]
Age category–from 36 to 45 years old	62	0.71	0.66	0.72	[0.12–4.42]
Age category–from than 46 to 55 years old	115	0.35	0.32	0.25	[0.59–2.07]
Age category–from than 56 to 65 years old	114	0.39	0.35	0.3	[0.06–2.28]
Age category–over 65 years old	63	0.26	0.24	0.15	[0.04–1.64]
Land type–hill	59				
Land type—Upland	180	0.81	0.298	0.57	[0.79–2.78]
Land type–Lowland	137	0.74	0.286	0.43	[0.35–1.58]

IT knowledge, use of smartphone to record information and intention to intensify production were positively associated with ‘practicality’ and ‘usefulness’ factors and negatively associated with ‘external pressure and negative feelings’ factor.

### 3.6. Association between use of EID technology and lameness levels

Farmers using EID technology (‘adopters’) for management from September 2014 to August 2015 had significantly lower flock lameness levels (median 5, IQR 2–6) compared to farmers who did not intend to adopt the technology (‘non-adopters’) (median 5, IQR 3–10) and farmers intending to adopt it in the future (‘intenders’) (median 5, IQR 4–10) (χ^2^ = 10.91) p = 0.005).

**Fig 4:**
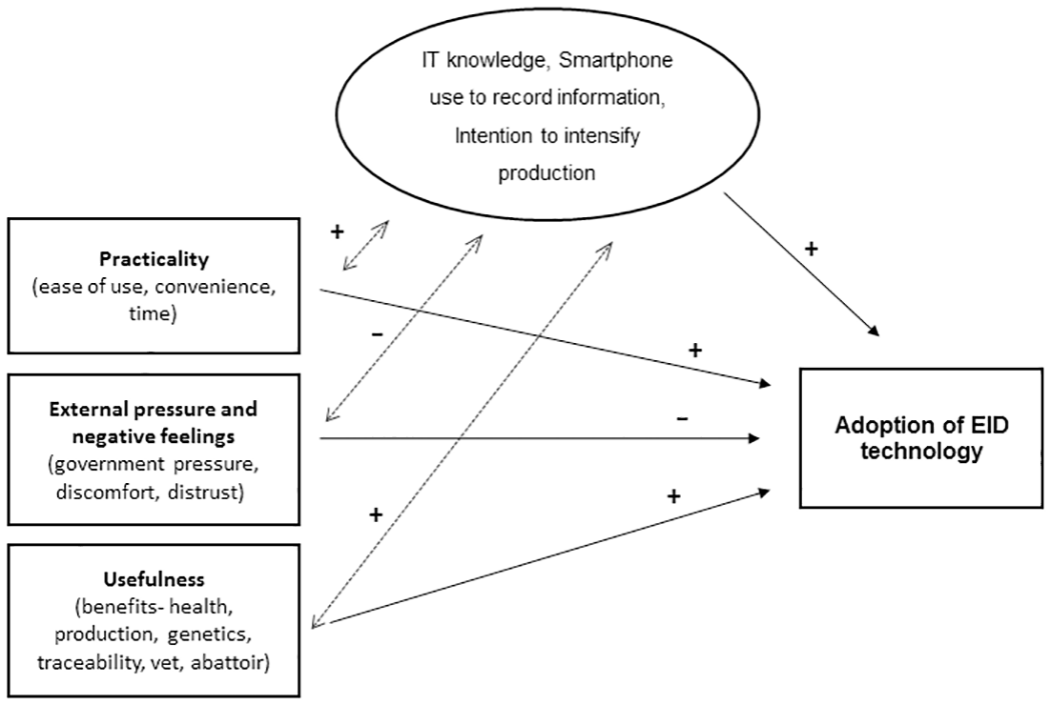
Framework obtained from results of this study with regards to factors associated with EID technology adoption (+ and–signs indicate direction of associations).

Fig 4 presents the framework obtained from our results. Farmers with high IT knowledge, using a smartphone to record information on farm, and with intention to intensify production were more likely to have adopted/intend to adopt EID tools to record flock information than farmers not having these characteristics. Farmers who had adopted /intended to adopt EID technologies were more likely to perceive it as practical and useful than non-adopters. On the contrary, external pressure and negative feelings factor was negatively associated with uptake of EID technologies.

## 4. Discussion

To the authors’ knowledge, this is the first study exploring farmer’s beliefs towards EID related technology. One of the key and novel findings in this study is that ‘external pressure and negative feelings’ factor seems to be significant in the adoption of technology, in addition to the practicality and usefulness aspects of technology—two constructs which are most frequently studied in technology adoption [32–34]. This factor included beliefs that negatively impacted adoption, that is, farmers that felt under pressure to adopt technologies were less likely to adopt EID recorded information for flock management. These farmers were more likely to see EID technology as an extra burden for farmers, complex, and had higher level of distrust and scepticism in current technology. This is consistent with the ‘Technology Readiness Index’ (TRI) paradigm, which argues that discomfort and insecurity towards a technology act as inhibitors of acceptance and have a negative relationship with technology adoption [19,24]. There is anecdotal evidence that legislation related to implementation of sheep EID in the UK was not well accepted among some farmers, who saw it as an extra bureaucratic burden with no clear benefits. This is also indicated by results in the current study as even though all farmers were complying with legislation by having EID tags for their flock, only 53% farmers were further utilising the presented opportunity to use EID technology for management by purchasing or owning EID readers. Furthermore, only 21% were actually using the EID technology for management purposes. This indicates that, despite investment, a low proportion of farmers are using this technology for management purposes.

There could be several factors explaining this. First, as mentioned above, legislation involving a mandatory aspect of EID tagging lacked an overall approval of the sheep industry which may have generated negative perceptions and exacerbated feelings of pressure among farmers, and contributed to reluctance in adopting any EID equipment for management. Science and technological innovations are shaped by the social and political context they are developed within [35]. People’s views on this social and political context influences their views of the technology [36,37]. Farmers feel that they are over burdened with regulations and audits from industry and government, and that mechanisms for auditing farmers are also often ineffective [38]. The correlation between farmer’s views that there is too much pressure on them to adopt new technologies and that EID adds to the complexity of their information gathering demands–factors that relate to the compulsory use of EID for traceability, and how likely they are to adopt the technology for their own management purposes suggests that some farmers are being influenced by what they perceive as the negative political connotations of EID. A recognition of farmer’s own forms of expertise and experience into the design of technologies [38] and measures to improve disease management [39] can foster trust and give farmers more ownership over disease management, rather than top down measures which farmers might find problematic. Similar approaches utilising principles of co-production have been used in health care for technology adoption [40].

Secondly, it is possible that feelings of external pressure further compounded by lack of published evidence and validated case studies on the beneficial effects of EID technology is responsible for generating negative feeling among farmers with regards to added complexity and distrust in technology. However, farmers who had better IT knowledge and were already using smartphone to record information were less likely to have these negative feelings and more likely to adopt technologies. This suggests that one way to negate these negative feelings might be by enhancing IT capability of farmers.

In the current study, two other factors–‘*practicality’* and ‘*usefulness’* were significantly associated with adoption of EID technology i.e. farmers that perceived EID related technology as useful and practical were significantly more likely to adopt or intend adopting it. These results are consistent with the “Technology Acceptance Model”, which argues that “perceived ease of use” and “perceived usefulness” are key predictors of technology adoption [32,41]. Previous research on the adoption of technologies in agricultural field has reported similar results [34,42]. The importance of designing technologies that are easy to use and useful for the farmers has been previously highlighted [38]. Messages focussing on beneficial effects and the ease of use of EID technology may strenghten technology uptake.

Cost of the technology was an important factor across all the groups (adopters, intenders and non-adopters) as only 9% farmers disagreed or strongly disagreed with cost as important. The cost of an EID reader will depend on the complexity and features of the model, and current prices vary between £300 and £1000 approximately. Lack of resources (financial or others) are a well know barrier for the adoption of PLF tools [42–45]. However, technology adoption decision is frequently reported to be influenced by an assessment of the ‘cost effectiveness’ of the tool [46,47] and for this reason it would be expected that all farmers would rate importance of financial cost for adoption decision highly along with productivity and time saving gains. These results suggest that both adopters and non-adopters consider the ‘absolute’ cost of the tools an important factor in the adoption decision, possibly due to the low profit margins in sheep farming seen in recent decades.

One interesting finding of this study was that farmers from both groups (non-adopters and adopters/intenders) tended to disagree with the statement “Adoption of EID by other farmers is important to my decision to use EID recording for farm management” (only 20% farmers agreed or strongly agreed) with no significant difference between groups) suggesting ‘social pressure’ is not influential in adoption. This contradicts the findings of Kutter et al., (2011), who collected farmer’s opinions about the use of PLF tools, and concluded that other farmers are regarded as very important for promoting interest in the topic. Other studies, however, pointed out that technology adoption is a highly individualistic process, conducted according to farmer’s personality and experience, among other factors [48], and this may explain results in the current study.

The most important farmer characteristics predicting adoption of EID recorded information for flock management were the farmer’s IT literacy and use of smartphone technology. This is not surprising, since PLF technologies are ‘data intensive’, and farmers with lower levels of IT literacy may struggle to manage and use efficiently big amounts of collected data [49,50]. Moreover, farmers already using technology (i.e., smartphone or computer) may find the introduction of new technology on farm compatible with existing practices. Compatibility with farming operations, equipment, and routines has been shown to have a significant effect on farmer perception of ease of use of technology, and indirectly on technology adoption [42].

Intention to increase production in the future was also significantly associated with adoption of EID related technology. Similarly, intensity of production was observed to be associated with adoption of precision farming technologies among Irish dairy farmers in a recent study [51]. This is in line with previous research indicating a relationship between adoption of new technologies by farmers and attitude towards investment and risk [45]. The proportion of labour time spent by a farmer in managing the flock in the previous year was positively and significantly associated with adoption of EID technology. This could be due to the fact that time spent could facilitate familiarity with technology which could then enhance confidence and influence perception of ease of use and perceived benefits. Off-farm employment has been negatively associated with the adoption of precision farming tools among US farmers due to lack of time to gain familiarity [52,53].

Our results show that other known sociodemographic factors seen as influencing technology uptake, such as age or enterprise size, did not significantly influence adoption of EID technology. Effect of age on adoption of technology has been variable with some studies suggesting this as a significant factor and poor adoption of technology with increasing age [54,55] whilst other suggesting age as not a barrier for adoption [42,53].

Previous research has also reported contradictory results with regards to enterprise size: while Aubert et al. (2012) reported no association between technology adoption and enterprise size, several other studies have reported a positive relationship [33,52,53,56,57]. It is important to emphasize that flocks in the current study were commercial breeding flocks with a median flock size of 500 which is larger than average flock size in the UK [58].

The use of EID technology for flock management was significantly associated with lower lameness levels. Lameness levels used in this study were estimated and reported by the farmers and fit closely to recent estimates of lameness prevalence [20]. Lower lameness levels could be due to the fact EID recorded information can be utilised to record individual sheep treatments and identify lame animal for isolation and culling, which is recommended best practice to reduce flock lameness levels [12,23]. Farmers using EID technology may also be more aware of the lameness levels of their flock, in contrast to farmers not using it. Farmers who rely on memory to identify sheep for culling have been previously reported to have higher relative risk of lameness [20]. All this suggests that EID technology could act as an important tool for management and control of lameness. Farmers always treating lame sheep with antibiotics (i.e following one of recommended practice to reduce lameness) [22] were also significantly more likely to be adopters of EID technology. This suggests that this group of farmers is perhaps more open to new innovations and have positive perceptions towards technology due to associated health and welfare benefits.

The selected sample for the survey was not random *per se* but the sample list had commercial farmers distributed across England and Wales and there was no difference between respondents and non-respondents with regards to location. There is still possibility that the results especially regarding absolute distribution of adopters and non-adopters are not representative of the whole of the UK or the entirety of England and Wales. However, this is less likely to affect the associations among the factors and adoption of EID technology. Despite this, the framework of factors associated with adoption of EID technology as presented in this study does not imply causation. The likely impact of these factors on adoption needs to be tested further in intervention studies and in confirmatory factor analysis.

One of the disadvantage of collecting data on by questionnaire on beliefs is that there may be a self-report bias. However, as recommended in the literature, actions were taken to reduce this bias and increase validity of the questions (i.e. phrasing belief statements in a non-judgmental way, and assurance that responses would remain confidential and anonymous). [59]. The results of the current study give us insight into what factors influence adoption of EID technology on farms and can be used to target actions to positively influence uptake by farmers. We believe our results also have a wider application to adoption of technology in general, and raise interesting questions on the inclusion of external pressures and negative feelings felt by farmers in adoption models. We need further work to explore how beliefs related to feelings of discomfort, distrust and external pressure are being formed in the farming community and investigate which specific functionalities of EID technology act as barrier for farmers (such as reading of the tags, use of software, or others) to further enhance adoption.

## 5. Conclusion

In this study English and Welsh sheep farmer’s perceptions and their underlying beliefs towards EID technology were captured for the first time, giving new insights into barriers and drivers of adoption of this kind of technology. We conclude that the adoption of EID technology is influenced by three correlated factors: ‘practicality’, ‘usefulness’ and ‘external pressure and negative feelings’. Well-communicated evidence of the positive effects of EID technology on farm performance and the health and welfare of the flock, co-production of EID technology service involving farmers, enhancing farmer’s capability in use of technology is likely to enhance both farmer’s trust in technology and its subsequent adoption. However, EID technology must be practical and cost effective. Factors such as age, farm type (upland or lowland) or size of farm seem to be less important for adoption of EID technology.

## Supporting information

S1 Questionnaire Questionnaire used in this study. (PDF)

S1 Dataset Questionnaire responses. (XLSX)

## References

[1] Redman G. John Nix Farm Management Pocketbook. 47th editi [Melton Mowbray]: Agro Business Consultants; 2016.

[2] DEFRA. Farm business income by type of farm in England, 2015/16 [Internet]. 2016. https://www.gov.uk/government/uploads/system/uploads/attachment_data/file/562946/fbs-businessincome-statsnotice-27oct16.pdf

[3] DEFRA. Farm Business Income by type of farm in England, 2014/15 [Internet]. 2015. https://www.gov.uk/government/uploads/system/uploads/attachment_data/file/471952/fbs-businessincome-statsnotice-29oct15.pdf

[4] DEFRA. Farm Accounts in England—Results of the Farm Business Survey 2013/14 [Internet]. 2014. https://www.gov.uk/government/uploads/system/uploads/attachment_data/file/385056/fbs-farmaccountsengland-11dec14.pdf

[5] Kaler J, Green L. Sheep farmer opinions on the current and future role of veterinarians in flock health management on sheep farms: A qualitative study. Prev Vet Med. Elsevier B.V.; 2013;112: 370–377. doi: 10.1016/j.prevetmed.2013.09.009 24120236 10.1016/j.prevetmed.2013.09.009 PMC3909466

[6] Croston D, Pollot G. Planned sheep production. 2nd ed Oxford: Blackwell Scientific Publications; 1994.

[7] Ait-Saidi A, Caja G, Salama AAK, Milán MJ. Implementing electronic identification for performance recording in sheep: II. Cost-benefit analysis in meat and dairy farms. J Dairy Sci. Elsevier; 2014;97: 7515–24. doi: 10.3168/jds.2014-8091 25282418 10.3168/jds.2014-8091

[8] Yule I, Eastwood C. Challenges and opportunities for precision dairy farming in New Zealand: Developing a research agenda to enhance farm management benefits from precision technology use [Internet]. 2011. http://www.oie.int/doc/ged/D13666.PDF

[9] Wathes CM, Kristensen HH, Aerts JM, Berckmans D. Is precision livestock farming an engineer’s daydream or nightmare, an animal’s friend or foe, and a farmer’s panacea or pitfall? Comput Electron Agric. 2008;64: 2–10. doi: 10.1016/j.compag.2008.05.005

[10] Berckmans D. Precision livestock farming technologies for welfare management in intensive livestock systems. Rev sci tech Off int Epiz. 2014;33: 189–196. doi: 10.20506/rst.33.1.2273 10.20506/rst.33.1.2273 25000791

[11] Banhazi TM, Black JL. Precision Livestock Farming: A Suite of Electronic Systems to Ensure the Application of Best Practice Management on Livestock Farms. Aust J Multi-disciplinary Eng. 2009;7: 1–13.

[12] Kaler J, Daniels SLS, Wright JL, Green LE. Randomized Clinical Trial of Long‐Acting Oxytetracycline, Foot Trimming, and Flunixine Meglumine on Time to Recovery in Sheep with Footrot. J Vet Intern Med. Wiley Online Library; 2010;24: 420–425. 10.1111/j.1939-1676.2009.0450.x 20051002

[13] Fishbein M, Ajzen I. Belief, Attitude, Intention and Behaviour: An Introduction to Theory and Research. Read MA AddisonWesley. 1975; 480 doi: 10.2307/2065853

[14] Davis F. A Technology Acceptance Model for Empirically Testing New End-User Information Systems. Massachusetts Institute of Technology 1985.

[15] Ajzen I. The theory of planned behavior. Orgnizational Behav Hum Decis Process. 1991;50: 179–211. doi: 10.1016/0749-5978(91)90020-T

[16] Ajzen I. Perceived Behavioral Control, Self-Efficacy, Locus of Control, and the Theory of Planned Behavior. J Appl Soc Psychol. 2002;80: 2918–2940. doi: 10.1111/j.1559-1816.2002.tb00236.x

[17] Ajzen I, Fishbein M. Attitude-behavior relations: A theoretical analysis and review of empirical research. Psychol Bull. 1977;84: 888–918. doi: 10.1037/0033-2909.84.5.888

[18] Rogers EM. Diffusion of innovations. Macmillian Publishing Co New York: The Free Press, A Division of Macmilan Publishing Co., Inc; 2003.

[19] Parasuraman A. Technology Readiness Index (TRI)—A Multiple-Item Scale to Embrace New Technologies. J Serv Res. 2000;2: 307–320. doi: 10.1177/109467050024001

[20] Winter JR, Kaler J, Ferguson E, KilBride AL, Green LE. Changes in prevalence of, and risk factors for, lameness in random samples of English sheep flocks: 2004–2013. Prev Vet Med. Elsevier B.V.; 2015;122: 121–128. doi: 10.1016/j.prevetmed.2015.09.014 26435034 10.1016/j.prevetmed.2015.09.014

[21] Kaler J, Green LE. Recognition of lameness and decisions to catch for inspection among sheep farmers and specialists in GB. BMC Vet Res. 2008;4: 41 doi: 10.1186/1746-6148-4-41 18854014 10.1186/1746-6148-4-41 PMC2588441

[22] Kaler J, Daniels SLS, Wright JL, Green LE. Randomized clinical trial of long-acting oxytetracycline, foot trimming, and flunixine meglumine on time to recovery in sheep with footrot. J Vet Intern Med. 2010;24: 420–425. doi: 10.1111/j.1939-1676.2009.0450.x 20051002 10.1111/j.1939-1676.2009.0450.x

[23] Wassink GJ, King EM, Grogono-Thomas R, Brown JC, Moore LJ, Green LE. A within farm clinical trial to compare two treatments (parenteral antibacterials and hoof trimming) for sheep lame with footrot. Prev Vet Med. Elsevier; 2010;96: 93–103. 10.1016/j.prevetmed.2010.05.006 20627343

[24] Godoe P, Johansen TS. Understanding adoption of new technologies: Technology readiness and technology acceptance as an integrated concept. J Eur Psychol Students. 2012;3: 38.

[25] Fabrigar LR, Wegener DT, MacCallum RC, Strahan EJ. Evaluating the use of exploratory factor analysis in psychological research. Psychol Methods. US: American Psychological Association; 1999;4: 272–299. doi: 10.1037/1082-989X.4.3.272

[26] Ferguson E, Cox T. Exploratory Factor Analysis: A Users’Guide. Int J Sel Assess. Blackwell Publishing Ltd; 1993;1: 84–94. doi: 10.1111/j.1468-2389.1993.tb00092.x

[27] Cronbach LJ. Coefficient alpha and the internal structure of tests. Psychometrika. 1951;16: 297–334. doi: 10.1007/BF02310555

[28] Tavakol M, Dennick R. Making sense of Cronbach’s alpha. Int J Med Educ. 2011;2: 53–55. doi: 10.5116/ijme.4dfb.8dfd 28029643 10.5116/ijme.4dfb.8dfd PMC4205511

[29] Distefano C, Zhu M, Mîndrilă D. Understanding and using factor scores: Considerations for the applied researcher. Pract Assessment, Res Eval. 2009;14: 1–11.

[30] Dohoo IR, Martin W, Stryhn HE. Veterinary epidemiologic research. 2003;

[31] Szumilas M. Explaining odds ratios. J Can Acad Child Adolesc Psychiatry. 2010;19: 227–229. 20842279 PMC2938757

[32] Davis F. A Technology Acceptance Model for Empirically Testing New End-User Information Systems. Massachusetts Inst Technol 1985; 291.

[33] Adrian AM, Norwood SH, Mask PL. Producers’ perceptions and attitudes toward precision agriculture technologies. Comput Electron Agric. 2005;48: 256–271. doi: 10.1016/j.compag.2005.04.004

[34] Flett R, Alpass F, Humphries S, Massey C, Morriss S, Long N. The technology acceptance model and use of technology in New Zealand dairy farming. Agric Syst. 2004;80: 199–211. doi: 10.1016/j.agsy.2003.08.002

[35] MacKenzie D, Wajcman J. Introductory Essay: The Social Shaping of Technology [Internet]. The Social Shaping of Technology. 1999.

[36] Irwin A, Michael M. Science, social theory and public knowledge. Irwin A, Michael M, editors. Maidenhead; 2003.

[37] Levidow L, Marris C. Science and governance. 2001;28: 345–360.

[38] Tsouvalis J, Seymour S, Watkins C. Exploring knowledge-cultures: Precision farming, yield mapping, and the expert—farmer interface. Environ Plan A. 2000;32: 909–924. doi: 10.1068/a32138

[39] Enticott G, Wilkinson K. Whose knowledge counts? Biosecurity socio-politics invasive species Infect Dis. Routledge; 2013; 91.

[40] May CR. Making sense of technology adoption in healthcare: meso-level considerations. BMC Med. 2015;13: 92 doi: 10.1186/s12916-015-0305-8 25902829 10.1186/s12916-015-0305-8 PMC4407548

[41] Davis FD, Bagozzi RP, Warshaw PR. User Acceptance of Computer Technology : A Comparison of Two Theoretical Models Author (s): Davis Fred D., Bagozzi Richard P. and Warshaw Paul R. Published by : INFORMS Stable URL : http://www.jstor.org/stable/2632151 REFERENCES Linked references ar. Manage Sci. 1989;35: 982–1003.

[42] Aubert BA, Schroeder A, Grimaudo J. IT as enabler of sustainable farming: An empirical analysis of farmers’ adoption decision of precision agriculture technology. Decis Support Syst. Elsevier B.V.; 2012;54: 510–520. doi: 10.1016/j.dss.2012.07.002

[43] Reichardt M, Jürgens C. Adoption and future perspective of precision farming in Germany: results of several surveys among different agricultural target groups. Precis Agric. 2009;10: 73–94. doi: 10.1007/s11119-008-9101-1

[44] DEFRA. Farm Practices Survey Autumn 2012 –England [Internet]. 2013. https://www.gov.uk/government/collections/farm-practices-survey

[45] Feder G. Farm Size, Risk Aversion and the Adoption of New Technology under Uncertainty. Oxf Econ Pap. 1980;32: 263–283. Available: https://www.jstor.org/stable/2662685

[46] Diekmann F, Batte MT. 2010 Ohio Farming Practices Survey: Adoption and Use of Precision Farming Technology in Ohio. Ohio State Univ Dep Agric Environ Dev Econ. 2010; http://static1.1.sqspcdn.com/static/f/891472/12275502/1305725357777/OSU+2010+2010+Ohio+farming+practices+survey+-+adoption+and+use+of+precision+farming+technology+in+Ohio.pdf.pdf?token=VvJ7a31RcGmyQARwpf3VjPMmOFs%3D

[47] Rehman T, McKemey K, Yates CM, Cooke RJ, Garforth CJ, Tranter RB, et al Identifying and understanding factors influencing the uptake of new technologies on dairy farms in SW England using the theory of reasoned action. Agric Syst. 2007;94: 281–293. doi: 10.1016/j.agsy.2006.09.006

[48] Alvarez J, Nuthall P. Adoption of computer based information systems—The case of dairy farmers in Canterbury, NZ, and Florida, Uruguay. Comput Electron Agric. 2006;50: 48–60. doi: 10.1016/j.compag.2005.08.013

[49] Fountas S, Wulfsohn D, Blackmore BS, Jacobsen HL, Pedersen SM. A model of decision-making and information flows for information-intensive agriculture. Agric Syst. 2006;87: 192–210. doi: 10.1016/j.agsy.2004.12.003

[50] Nash E, Dreger F, Schwarz J, Bill R, Werner A. Development of a model of data-flows for precision agriculture based on a collaborative research project. Comput Electron Agric. 2009;66: 25–37. doi: 10.1016/j.compag.2008.11.005

[51] Läpple D, Holloway G, Lacombe DJ, O’Donoghue C. Sustainable technology adoption: a spatial analysis of the Irish Dairy Sector. Eur Rev Agric Econ. 2017; 1–26.

[52] Daberkow SG, McBride WD. Socioeconomic Profiles of Early Adopters of Precision Agriculture Technologies. Journal of Agribusiness. 1998 pp. 151–168.

[53] Daberkow SG, McBride WD. Farm and Operator Characteristics Affecting the Awareness and Adoption of Precision Agriculture Technologies in the US, 163–177. Precis Agric. 2003; 163–177. https://link.springer.com/article/10.1023%2FA%3A1024557205871?LI=true

[54] Corner-Thomas RA, Kenyon PR, Morris ST, Ridler AL, Hickson RE, Greer AW, et al Influence of demographic factors on the use of farm management tools by New Zealand farmers. New Zeal J Agric Res. 2015;58: 412–422. doi: 10.1080/00288233.2015.1063513

[55] Warren M. Drivers and impediments in adoption of Internet in UK agricultural businesses. J Small Bus Enterp Dev J. 2016;11: 371–381. doi: 10.1108/14626000410551627

[56] Isgin T, Bilgic A, Forster DL, Batte MT. Using count data models to determine the factors affecting farmers’ quantity decisions of precision farming technology adoption. Comput Electron Agric. 2008;62: 231–242. doi: 10.1016/j.compag.2008.01.004

[57] Kutter T, Tiemann S, Siebert R, Fountas S. The role of communication and co-operation in the adoption of precision farming. Precis Agric. 2011;12: 2–17. doi: 10.1007/s11119-009-9150-0

[58] AHDB. UK Yearbook 2016—Sheep [Internet]. 2016. http://beefandlamb.ahdb.org.uk/wp/wp-content/uploads/2016/07/UK-Yearbook-2016-Sheep-050716.pdf

[59] Horne R, Weinman J. Patients’ beliefs about prescribed medicines and their role in adherence to treatment in chronic physical illness. J Psychosom Res. 1999;47: 555–567. doi: 10.1016/S0022-3999(99)00057-4 10661603 10.1016/s0022-3999(99)00057-4

