# Supplementary material for: Drivers for precision livestock technology adoption: A study of factors associated with adoption of electronic identification technology by commercial sheep farmers in England and Wales
Source: PLoS One. 2018 Jan 2;13(1):e0190489. doi: 10.1371/journal.pone.0190489 (PMC5749824; doi:10.1371/journal.pone.0190489)
Supplement: S1 Questionnaire — (PDF) [file pone.0190489.s001.pdf]

The University of Nottingham
UNITED KINGDOM · CHINA · MALAYSIA

Lameness management and recording on farm: Your decisions and opinions

Section 1. Information about you and your farm

1. How many years have you been farming sheep?_____**years**

2. In which age category are you?

   Unwilling to say☐ **Less than 25**☐ **26-35**☐ **36-45**☐

   46-55☐ **56-65**☐ **Over 65**☐

3. What digital technology and other devices do you use at home? (Please tick all that apply)

   Smartphone (iphone)☐ **Smartphone (android)**☐ **Computer**☐ **Tablet**☐
   Others (please specify)________________________________

4. What digital technology and other devices do you use on farm? (Please tick all that apply)

   Smartphone (iphone)☐ **Smartphone (android)**☐ **Computer**☐ **Tablet**☐

   EID reader☐ **Others** (please specify)_______________________

5. How would you describe your knowledge of IT? (Please tick one answer)

   Nil☐ **Low**☐ **Medium**☐ **High**☐

6. Which of the following do you use the internet for? (Please tick all that apply)

   Don't use☐ **email**☐ **Web browsing**☐ **Facebook**☐ **Twitter**☐ **Other** ☐

7. Which other enterprise do you have on farm? (Please tick all that apply)

   Beef☐ **Arable**☐ **Dairy**☐ **Others** (please specify)_____________

8. What percentage of your work time did you spend managing your sheep enterprise between Sep 2014-Aug 2015? ________**%**

9. How many other people worked (paid or unpaid) with your sheep flock between Sep 2014-Aug 2015? **Full time** _______and **part time** ________

10. What type of land was the majority of your farm between Sep 2014-Aug 2015? (Please tick one answer)

Hill☐ **Upland☐** **Lowland☐**

Section 2. Your flock production

11. How many ewes did you have in your breeding flock between Sep 2014-Aug 2015? _______**ewes**

12. What was your scanning percentage between Sep 2014-Aug 2015? _____**%**
(No. of lambs/100 ewes put to the tup)

13. How many lambs were sold, retained as replacements or as stores between Sep 2014-Aug 2015?
_____**Lambs sold** _____**Lambs retained as replacements** _____ **Lambs retained as stores**

14. In what month did you start lambing in 2015? ________

15. Did you house your sheep between Sep 2014-Aug 2015? (Please tick one answer)

Yes, ewes☐ **Yes, finishing lambs☐** **Yes, both☐** **No☐**

16. How many batches were lambing ewes separated into between Sep 2014-Aug 2015? (Please tick one answer)

1☐ **2☐** **3☐** **4☐** **5 or more☐**

17. How many ewes did you cull between Sep 2014-Aug 2015? ________

18. What were the main reasons for culling ewes between Sep 2014-Aug 2015? (Please tick all that apply)

Low productivity☐ **Poor condition ☐** **Tooth loss ☐** **Lameness☐** **Mastitis ☐**

Infertility ☐ **Other diseases** (please specify)__________________________

19. Have you made any changes to your sheep farming business between Sep 2014-Aug 2015? (Please tick all that apply)

No☐ **Yes, intensify☐ production** **Yes, extensify☐ production** **Yes, increase☐ flock numbers** **Yes, reduce☐ flock numbers**

20. Do you intend to make changes to your sheep farming business within the next two years? (Please tick all that apply)

No☐ **Yes, intensify☐ production** **Yes, extensify☐ production** **Yes, increase☐ flock numbers** **Yes, reduce☐ flock numbers**

Section 3. Lameness in your flock

Please answer the following questions about **lameness in your ewe flock for the period between Sep 2014 and Aug 2015.**

21. Please enter the **approximate percentage of lame ewes at the worst affected time point**, and of those, the ewes lame with footrot, scald and CODD for each quarter in the table below. There is one example provided.

Month	Total percentage lame ewes	Percentage lame with Footrot	Percentage lame with Scald	Percentage lame with CODD
**For example Month-13**	25%	10%	10%	5%
**Sep 14 to Nov 14**				
**Dec 14 to Feb 15**				
**Mar 15 to May 15**				
**Jun 15 to Aug 15**				

22. How soon did you treat individual lame sheep after seeing that they were lame, between Sep 2014-Aug 2015?

The first day you saw them ☐   **Within 3 days ☐**   **Within 1 week ☐**   **Within 2 weeks ☐**   **Longer than 2 weeks ☐**

Did not treat any individual lame sheep☐

23. How often did you use an antibiotic injection to treat individual lame sheep between Sep 2014-Aug 2015?

Always ☐   **Usually ☐**   **Sometimes ☐**   **Never ☐**

24. Did you select animals to cull based on lameness between Sep 2014-Aug 2015?

Yes ☐   **No ☐**

25. If yes, how many times did you treat an animal before selecting for culling?

1☐   **2☐**   **3☐**   **4 or more☐**

Section 4. Recording information on farm (EID)

26. How do you currently record information on farm? (Please tick all that apply)

Any piece of paper☐ Notebook/Diary☐ File card☐ Smartphone☐ PDA☐

Tablet☐ Computer☐

27. What kind of electronic identification device (EID) did you use between Sep 2014-Aug 2015?

Tags ☐ Boluses ☐ Both ☐

28. What kind of reader do you have?

Handheld ☐ Static ☐ None☐

29. Did you use EID recording for farm management purposes (e.g. sorting animals into groups, recording treatments, breeding information etc.) between Sep 2014-Aug 2015 ? (please tick all that apply)

Yes for Ewes ☐ Yes for Lambs☐ No☐ If "No "please go to question 33

30. When did you first start using EID recording for management purposes?
For ewes ______ (year) **For lambs** ______ (year)

31. Did you use any commercial farm management software packages between Sep 2014-Aug 2015?

Yes ☐ No ☐ If "No", please go to question 33

32. How easy is your farm management software package to use?
(Please tick only one answer)

Not easy at all	Not very easy	Unsure	Quite easy	Very easy
☐	☐	☐	☐	☐

33. If you do not currently use EID recording for management, do you intend to adopt it within the next year?

Yes ☐ No ☐ Not sure ☐ Not Applicable☐

34. How useful do you think EID recording is for management? (Please tick one answer)

Not useful at all	Not very useful	Unsure	Quite useful	Very Useful
☐	☐	☐	☐	☐

Section 5. Your opinions on data recording

35. For each statement below please indicate the extent to which you agree or disagree that the factor listed is important to **YOUR decision to USE EID recording for farm management purposes**:

	Disagree strongly	Disagree	Neither agree nor disagree	Agree	Agree strongly
The **ease of use** of EID technology is important to my decision to use EID recording for farm management	☐	☐	☐	☐	☐
The **time required to use** EID is important to my decision to use EID recording for farm management	☐	☐	☐	☐	☐
The **convenience of using** EID is important to my decision to use EID recording for farm management	☐	☐	☐	☐	☐
**Improvements in sheep health** resulting from using EID are important to my decision to use EID recording for farm management	☐	☐	☐	☐	☐
**Improvements in flock productivity** resulting from using EID are important to my decision to use EID recording for farm management	☐	☐	☐	☐	☐
The **cost** of EID equipment is important to my decision to use EID recording for farm management	☐	☐	☐	☐	☐
Adoption of EID by **other farmers** is important to my decision to use EID recording for farm management	☐	☐	☐	☐	☐
The fact EID is a **compulsory** technology is important to my decision to use EID recording for farm management	☐	☐	☐	☐	☐

	Disagree strongly	Disagree	Neither agree nor disagree	Agree	Agree strongly
**External support with the technology** is important to my decision to use EID recording for farm management	☐	☐	☐	☐	☐
The fact EID technology should allow me **to get more out of the veterinary consultation** is important to my decision to use EID recording for farm management	☐	☐	☐	☐	☐
The fact EID technology **should make it easier to receive information from the abattoir** is important to my decision to use EID recording for farm management	☐	☐	☐	☐	☐
The fact that EID technology helps with **animal traceability** is important to my decision to use EID recording for farm management	☐	☐	☐	☐	☐
The fact EID technology helps with **genetic selection, genealogy and crossbreeding** is important to my decision to use EID recording for farm management	☐	☐	☐	☐	☐
Fears about **technology failure and reliability** issues are important to my decision to use EID recording for farm management	☐	☐	☐	☐	☐
Finding an appropriate **management software package** that meets my requirements is important to my decision to use EID recording for farm management	☐	☐	☐	☐	☐

36. For each statement below about **EID technology in relation to farmers and the farming industry as a whole,** please indicate the extent to which you agree or disagree

	**Disagree strongly**	**Disagree**	**Neither agree nor disagree**	**Agree**	**Agree strongly**
EID assisted recording technology **adds to the complexity of information demands** placed on farmers	☐	☐	☐	☐	☐
EID assisted data recording is **less stressful for the stock** than having to manually read ear tags	☐	☐	☐	☐	☐
Increased technology adoption and use of precision farming is **beneficial** for the farming industry	☐	☐	☐	☐	☐
There is **too much pressure** on farmers by the government and the market to adopt new technologies	☐	☐	☐	☐	☐
Current technology for data recording is **not 'future proof'**, hence it is better to wait longer before making an investment	☐	☐	☐	☐	☐
Farmers **need more support** developing the skills to use EID assisted data recording technologies effectively	☐	☐	☐	☐	☐

Thank you very much for your participation
